# Implementation of the Sono-Heat-Exchanger in the Extra Virgin Olive Oil Extraction Process: End-User Validation and Analytical Evaluation

**DOI:** 10.3390/molecules24132379

**Published:** 2019-06-27

**Authors:** Lorenzo Cecchi, Maria Bellumori, Filomena Corbo, Gualtiero Milani, Maria Lisa Clodoveo, Nadia Mulinacci

**Affiliations:** 1 Department of NEUROFARBA, and Multidisciplinary Centre of Research on Food Sciences (M.C.R.F.S.- Ce.R.A.), University of Florence, Via Ugo Schiff 6, 50019 Sesto F.no Firenze, Italy; 2 Department of Pharmacy-Pharmaceutical Sciences, University Aldo Moro-Bari, Via Orabona, 4, 70100 Bari, Italy; 3 Interdisciplinary Department of Medicine, University of Bari, Piazza Giulio Cesare, 11-70124 Bari, Italy

**Keywords:** ultrasounds, end-user validation, hydroxytyrosol, phenolic compounds, volatile compounds, HS-SPME-GC-MS, HPLC-DAD, oil yields

## Abstract

The use of innovative systems, such as the heat exchanger, for production of extra virgin olive oil should allow maintenance of the same quality of those oils derived from traditional processes, and presents specific advantages. The performance of this system was evaluated by (i) determining the parameters directly measurable by the olive millers (i.e., end-user validation based on the production yields when the plant is located in different processing lines) and (ii) assessing the product quality through estimation of the content of phenolic and volatile compounds. The phenols were determined by High Performance Liquid Chromatography with Diode Array Detector (HPLC–DAD) before and after acidic hydrolysis while the volatile fraction was studied by Head-Space Solid-Phase-Micro-Extraction Gas-Chromatography with Mass Detector (HS–SPME–GC–MS). The use of the sono-heat-exchanger presents several advantages: it is a flexible machine, able to interface with all devices of the world’s leading manufacturers of the Extra Virgin Olive Oil (EVOO) extraction plant, and it guarantees shorter processing times and energy savings. Our results also pointed out its capability to increase the oil yields up to 5.5%, particularly when it extracts oil from unripe fruits, which in traditional processes yield oils with higher phenolic contents, but with lower oil yields. Overall, the quality of virgin olive oils was maintained, avoiding decreases of phenolic content or detrimental effects on the sensory characteristics.

## 1. Introduction

The involvement of end-users, namely the olive millers, in the development of innovative mechanical devices is fundamental in order to accelerate the transfer of research results and determine a change to the production system of extra virgin olive oil (EVOO) [1]. Indeed, only after process qualification has been completed can the manufacturing process begin production for commercial use [2]. Process qualification is the second stage of process validation [3] and concerns the qualification of manufacturing and production processes to confirm they are able to operate at a certain standard during sustained commercial manufacturing. The process validation of a technology represents an important step to move from an innovative idea to the diffusion of the new technology and its application in the reference-manufacturing context through a validation process. The purpose of the validation is to provide documented evidence to demonstrate with confidence that a specific process allows the constant production of a product that meets its predefined specifications and quality attributes [4].

Process qualification of industrial implementation of the sono-heat-exchanger in the EVOO extraction process has been discussed partially in previous papers covering the following aspects of manufacturing: (i) consumption of direct material, (ii) production times [5], (iii) labor [6], job security [7], (iv) occupied spaces [8], (v) energy consumption [9], (vi) investment/plant management costs [10], (vii) decrease in lead time, i.e., the time taken by the material to enter the raw warehouse and exit the finished warehouse [11].

The experimental phase of this work consists of the qualification of the processes aimed to cover other aspects of process qualification of the industrial implementation of the sono-heat-exchanger in the EVOO extraction process: (i) quality and stability of the process and (ii) higher and constant quality of the finished product.

During this phase, it is important to determine if the process is able to produce, in a reproducible way, a high amount of high quality EVOO through the simultaneous administration of both ultrasonic and thermal energy to the olive paste.

The objective of measuring of the performance of the innovative system was achieved through two strategies: (i) evaluating the results directly perceptible and measurable by the olive millers (i.e., end-user validation based on the estimate of production yields when the plant is located in processing lines that differ in brand, technology, and work capacity, using olives characterized by different cultivars and maturation indices); (ii) assessing the product quality through estimation of the content of phenolic and volatile compounds. Achieving these two objectives required a multidisciplinary approach [12].

The interdisciplinary research activity is essential to transfer to the olive oil industry the most recent technologies useful for supporting the income of the millers, increasing simultaneously the quantity of extractable oil from the olives and its quality in terms of concentration of healthy molecules with different pharmacological profiles [13].

The implementation of the first industrial-scale plant prototype in the olive oil extraction process combines two technologies: the ultrasounds and the heat exchanger, which is able to heat and cool oil pastes. This prototype is characterized by a high level of technological maturity (TRL, technology readiness levels), i.e., 7, which corresponds to the development of the system and which must conduct its validation based on an end-user validation protocol. This latter step is crucial as it allows the researcher not only to understand if the innovation meets the real needs of those who will be its users (the millers), but also to have useful opinions for optimization directly from the insiders of a specific sector [14].

End-user validation is the best strategy to transform a technology into something that really meets the needs of the olive oil sector, determining the desired changes in the value of the product (i.e., a hydroxytyrosol content coherent with the application of health claims approved by the European Food Safety Authority (EFSA)) and in the income of the producers (due to the premium price gained by the high quality market segment) [15,16].

Technology validation and technology transfer are indispensable factors for ferrying modern mills towards greater competitiveness in the global market based on two fundamental principles: producing higher quantities of oil and obtaining products of superior quality [17,18]. The achievement of the TRL 9, i.e., the technology in its final form, still requires some efforts that are only possible with the collaboration of the millers, who are able, from the height of their experience, to suggest ways of continuous improvement also in relation to the organoleptic characteristics desired in the product.

The aim of the work was to validate the application of ultrasound in the EVOO extraction process in order to ascertain if, when the system prototype is placed in the operational environment, the innovative method is able to increase oil yields, reduce the process time, and guarantee oil quality preservation. Several batches of olives of several cultivars, grown in different geographical areas from the Apulia region in 2017 and harvested at different maturation indexes, were selected to produce the 23 oils analyzed in this work. The analytical investigations were applied to all the EVOOs produced from the same batches of olives, with and without the use of ultrasounds. In particular, for the analysis of phenolic profiles, we used the official method of the International Olive Council (IOC) [19] improved by acid hydrolysis of the hydroalcoholic extract [20,21] and recently validated after these modifications [22,23]. The analysis of volatile compounds by HS–SPME–GC–MS [24] has been performed in order to assess if the introduction of ultrasound can induce a damage of the enzymatic complex of the lipoxygenase (LOX) pathway with a detrimental effect on the sensory characteristics of the product.

## 2. Results and Discussion

### 2.1. Usability of the Sono-Heat-Exchanger (SHE) and Oil Yields

**Figure 1:**
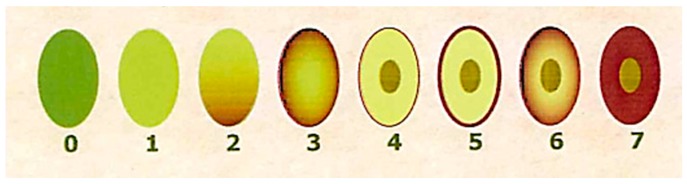
Ripening index according to the different color of the fruit.

Prototype development requires identification and control of risks that arise from a specific context of use and the characteristics of the user (for example level of education, and intellectual and technical skills). In order to demonstrate that these requirements are satisfied, it is necessary to validate usability. The IEC (International Electrotechnical Commission) 62,366 and the Human Factors Engineering Guidance Document of the FDA [25] provide guidance on how this validation must be carried out. It is required that the representative users (olive millers) are involved and the protocol is applied in its representative context of use (usage environment; the olive mill). Indeed, it is necessary to run through the core tasks within a participatory observation of genuine users in an actual or simulated user environment and let the security functions run. Only when users are able to achieve the targets, thus proving that user requirements are met, is the usability of product validated. To this aim, three different olive millers and mills were selected to test the performance of the sono-heat-exchanger (SHE). The oils were produced by processing olive fruits of cultivars collected from several geographical areas in the Apulia region, and with a different maturity index, according to data shown in Table 1 and Table 2 and in Figure 1.

**Figure 2:**
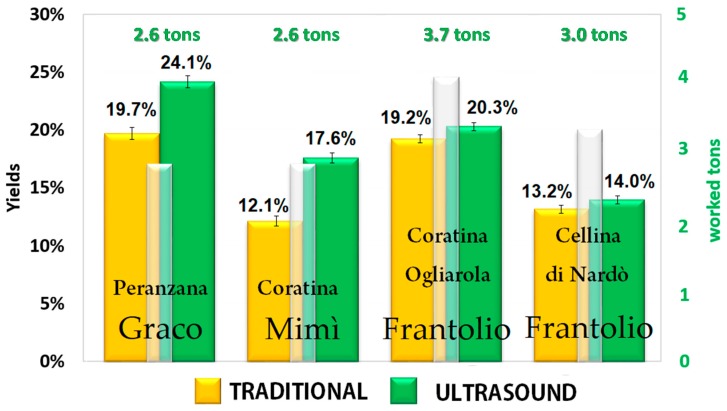
Mean oil yields obtained in the different oil mills, according to Table 1. The white bars indicate the tons of milled olives; the yellow and green bars indicate the oil yields obtained with and without the use of the sono-heat-exchanger (SHE), respectively.

The SHE is essentially a piping through which the olive paste passes. If the heat pump and the driver are turned off, it is a simple through pipe, while, if they are turned on, the olive paste is simultaneously subjected to the exchange of thermal and ultrasonic energy. Therefore, in order to assess the effect of the innovative SHE system on the quantity and quality of the extracted EVOO, batches of olives were selected considering cultivar, harvesting area, and ripeness index. Each batch was divided into identical portions and processed both with and without ultrasounds, at a temperature of 24 °C, according to a randomized scheme. The malaxation was not applied when the SHE turned on and this part of the plant was used only to transit the product into the decanter. The oil yields were calculated as the difference from the total oil content in the fruit and the total oil content in the olive pomace; it ranged from 12.1% to 24.1% depending on the cultivar and the maturity index of the olives, as shown in Figure 1. When applying ultrasound during milling, the oil extraction yields were always higher than those derived by the traditional process. Furthermore, combining the results shown in Figure 2 and the maturity index in Table 1, it is clear that the positive effect of the ultrasound treatment on oil yields was more evident when the olives were unripe than after fruit ripening. Indeed, it is more difficult to extract oil from green firm olives since the oil is contained in the cell vacuoles, which are not so easy to rupture. Ripening of fleshy olive fruit is one of the lasts developmental phases of fruit ontogeny, and it involves many changes including the accumulation of pigments and sugars and the process of flesh softening allowing an easier release of oil from very ripe fruits. Another interesting aspect is that the oils extracted from green fruits contain higher amounts of phenolic compounds, which increase the shelf life and the health quality of the EVOO, also increasing its commercial value. Having available a technology that allows the miller to anticipate the harvest without penalizing yields, reducing production times and obtaining an oil with a higher market value, contributes to increased profits and improved income. It has been previously demonstrated the use of the sono-heat-exchanger during milling allows energy savings [1,9].

### 2.2. Determination of Phenolic Compounds

Each mill produced several batches of oils both with and without applying the SHE (Table 1). After evaluation of oil yields, the phenolic and volatile fractions of samples were analyzed and the sensorial characteristics were evaluated by expert panelists (data not shown), to confirm they belonged to the extra virgin category. These analyses were selected for defining the final quality of the produced oils.

**Figure 3:**
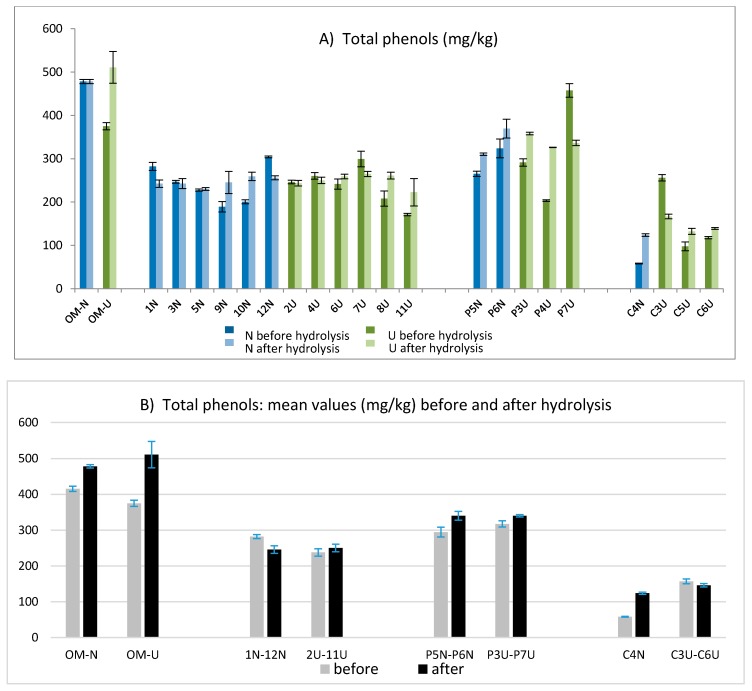
Total phenols obtained before and after the acidic hydrolysis for each EVOO (**A**) and averaged over all oils obtained in each oil mill (**B**).

The total phenolic amount was determined according to the IOC method and then the sum of hydroxytyrosol and tyrosol was evaluated after acidic hydrolysis applying the validated method [23]. The values obtained before and after hydrolysis are compared in Figure 3A, while the mean values obtained for each group of oils from different mills are compared in Figure 3B. As expected, the richest variety in terms of phenolic compounds was Coratina (samples OMU and OMN) followed by the Coratina–Ogliarola blend (samples P6N and P7U), while the lowest values were obtained for oils derived by Cellina di Nardò (C3U, C6U, C5U, and C4N). Overall, the values of total phenols varied in a wide range, showing concentrations from 58 mg/kg to 510 mg/kg. For fresh EVOOs, the amount of free hydroxytyrosol and tyrosol was negligible in most of the samples, while an average concentration of 14.5 mg/kg was observed only for samples from Coratina and the Coratina–Ogliarola blend (data not shown).

The hydrolysis was applied to obtain a more accurate determination of the total tyrosol and hydroxytyrosol (free and linked forms) derived from their secoiridoidic precursors [23]. This approach, already applied in previous works [20,21,22], helped to better estimate the antioxidant potential of the analyzed oils. Comparing the amount of total phenols obtained before and after hydrolysis, the main differences were highlighted only for few samples: OMU, P4U, P7U, and C3U (Figure 3A). Data obtained before and after the hydrolysis showed a wide variability in terms of total phenols, ranging from values lower than 100 mg/kg (C4N before hydrolysis) to values higher than 500 mg/kg (OMU after hydrolysis). The results in Figure 3B pointed out that no significant differences were found between the EVOOs obtained from a traditional technology (N) and those obtained by the help of ultrasounds (U).

**Figure 4:**
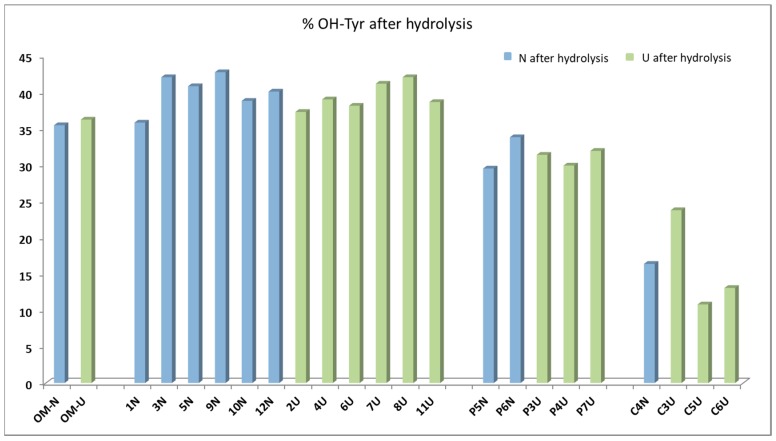
Hydroxytyrosol percentages evaluated after the acidic hydrolysis for the 23 oils and expressed on the sum of tyrosol and hydroxytyrosol.

Figure 4 shows the percentages of hydroxytyrosol in the sum of tyrosol and hydroxytyrosol. It emerges that the percentages of hydroxytyrosol ranged from 30% to 45% for most of samples, with exceptions only pointed out for oils from Cellina di Nardò cv (C4NI, C5UI, C3UI, C6UI), which showed lower percentages (10%–25%). Unlike expected, the results obtained after hydrolysis suggested that the main secoiridoidic components of the virgin olive oils were not oleuropein derivatives, but ligstroside analogues, which contain tyrosol in their structures. Recent findings, obtained by applying the same hydrolytic method on virgin oils from various provenance and varieties [22,23], highlighted that the most of oils showed hydroxytyrosol below 50%, confirming the prevalence of tyrosol precursors. In another study, it was proposed to sum the tyrosol derivatives and the hydroxytyrosol ones in order to evaluate the total useful phenolic content to apply the EFSA health claims [26]. The application of the acidic hydrolysis on a larger number of oils will help to acquire a more realistic picture of the secoiridoid pool in virgin olive oils.

### 2.3. Effect of US on Volatile Organic Compounds

The sensorial properties of virgin olive oils are crucial for defining the quality of the product [24], thus the content of the volatile organic compounds (VOCs) present in the headspace of our samples was determined by HS–SPME–GC–MS. Table 3 shows the content of two groups of VOCs in our samples, selected according to the literature: the first group (first 5 rows in Table 3) is constituted of the molecules originating in the LOX pathway, which are typically associated with the green and fruity notes of virgin olive oils (positive attributes); the second one (last 3 rows in Table 3) is represented by the C7 to C10 aldehydes, both saturated and unsaturated, which are linked to oxidative defects of virgin olive oils [27].

Overall, the total amount of the molecules originating in the lipoxygenase (LOX) pathway (green and fruity notes of virgin olive oils (VOOs)) is not significantly affected by the application of the ultrasound technology. At the same time, the different classes of VOCs belonging to the LOX cascade are not affected by the application of such technology. Furthermore, no increase of saturated and unsaturated aldehydes was observed. All these data confirm that the ultrasound technology does not affect the volatile profile of the obtained virgin olive oil samples.

On the contrary, even if our experiments are not aimed at highlighting differences due to the cultivar, data in Table 3 clearly confirm that the cultivar affects the concentration of these molecules in the oils. The monocultivar oils obtained from Coratina show contents of molecules from the LOX pathway (up to 38 ppm) at least three times higher than the other cultivars, with Cellina di Nardò showing the lowest content (approx. 6 ppm). Regarding aldehydes, all cultivars showed negligible amounts of the monounsaturated ones (which are more linked to the oxidative defects), while Ogliarola and Coratina showed amounts of saturated and di-unsaturated aldehydes slightly higher than Peranzana and Cellina di Nardò, with, however, very low amounts.

### 2.4. Panel Test

During this study, the sensorial quality of oil samples was also evaluated by a panel of expert panelists, not only to detect the possible defects as requested by the official procedure to classify the virgin olive oils, but mainly to collect information on specific positive notes.

The overall shape of the product sensory fingerprint is globally unchanged in samples obtained with or without ultrasound (Figure S1), except for the differences in the intensity of the fruity (higher in the sonicated sample) as well as for the perceptions of tomato, almond, artichoke, and leaf. The evaluation of bitterness and pungency, typically linked to the presence of phenolic compounds [27], allowed pointing out that the samples obtained with the aid of ultrasound (US) show a bitterness intensity of 20% higher than the traditional ones; these latter, on the contrary, are characterized by a greater perception of pungency. Overall, the samples obtained after ultrasound treatment have been defined as more harmonious than the non-sonicated ones by the panel group. The greater intensity of olfactory perceptions is indirect proof of the fact that the mechanical action of cavitation does not damage the enzymatic pathway of the LOX responsible for the positive characteristics of the product. The outcome of the evaluation of taste perceptions (bitterness and pungency) is the signal that the sono-chemical action influences the classes of phenolic compounds that are transferred from the oil paste to the lipid phase.

## 3. Materials and Methods

### 3.1. Visual Determination of the Maturity Index (MI)

The MI was determined during sampling by a panel of experienced evaluators, who assessed the skin and flesh color of the olives according to previous works [28]. Briefly, olives were divided into eight groups according to the characteristics summarized in Table 2 and Figure S1. The official method uses 100 olives and calculates an overall MI value for each sample or group of olives. The MI is calculated using the following equation:

MI = ∑_i_ × n_i_/100 (1)

where i is the group number and n_i_ is the number of olives in the group.

Olives were examined by manual cutting of pulp, and then counting the ones belonging to each category (Table 2, Figure 1). The method also involves calibrating other measurements of the fruit, such as weight, size, and diameter.

### 3.2. Oil Yield Evaluation

The oil yield was expressed as percentage of olive oil obtained by processing 100 kg of olives. It was evaluated by comparing the total oil content of the fruit (on a fresh matter basis) to the oil residue in the olive pomace derived from both the traditional and innovative extraction systems. The fruit and the pomace oil content (on fresh weight) has been determined in triplicate for each sample by the Soxhlet method [29]. The data are summarized in Figure 2.

### 3.3. Oil Production and End-User Validation Protocol

The oils were produced during the 2017–2018 crop seasons in industrial plants producing olive oil (1500–3000 kg/h) located in three different geographical areas of the Apulia region (Frantoio Mimì, Az. Agricola Donato Conserva Modugno (BA); Frantoio Graco Food, Torremaggiore (FG); Il Frantolio–Cisternino, Ostuni). The extraction plants of the all three mills were provided with a two-phase centrifugal system for oil separation. Two of them were equipped with a hammer crusher, while the third one (Frantoio Graco Food) was also provided with a de-stoner. All the plants had the malaxer hermetically closed, thus reducing the contact the olive paste with oxygen.

The homogeneous batches of olives (cv Coratina, Peranzana, Ogliarola, and Cellina di Nardò) were divided into samples of 800 kg each. Olives were washed before crushing. The crushed olive paste was then moved into the sono-heat-exchanger (SHE) and then it reached the malaxer. The SHE was characterized by a work capacity of 1500 kg/h, and it was equipped with 56 transducers (100 watt and 31 kHz) able to transfer a specific energy equal to 18,000 kJ/kg. Moreover, the SHE was also used to keep the temperature constant (25 °C), thanks to its capability to cool and heat the olive paste. The different olive production lots were treated alternately with or without ultrasounds (i.e., the SHE was activated or not) in order to compare the effect of ultrasounds with respect to the traditional process. After application of the SHE, the malaxer was used as a buffer to continuously feed the decanter; the malaxation time was set at 0 min for the sonicated samples and at 30 min for the traditional ones. The resulting EVOO was collected, filtered, weighed (to determine the yields), and stored in dark bottles (500 mL) at 15 °C until chemical analysis. A total of 23 samples (Table 1) were obtained and analyzed.

### 3.4. Evaluation of the European Legal Quality of the Virgin Olive Oils

Free acidity (% oleic acid), peroxide value (meq_O2_/kg), and spectroscopic indices were measured according to the analytical methods described in European regulation 2568/91 and the next amendments and additions [30]. According to the official method (European regulation 2568/91, annex XII, and subsequent amendments), the organoleptic evaluations were carried out by the professional panel group of the University of Bari (panel leader Dr. Alfredo Marasciulo), composed of 11 professional tasters, trained to recognize, describe, and quantify basic taste and odor properties. Olive oils were described through both positive (“fruity”, “bitter”, and “pungent”) and negative (for instance “rancid”, “fusty”, “musty”, and “winey”) attributes.

### 3.5. HPLC–DAD Analysis of the Phenolic Compounds before and after Acidic Hydrolysis

Phenolic compounds from olive oils were extracted and analyzed according to the IOC official method [19]. Phenolic compounds were extracted with a MeOH:H_2_O 80:20 *v*/*v* solution and immediately analyzed by an HP1100 system equipped with an autosampler, a column heater module, and a quaternary pump, and coupled to a diode array detector (DAD) (all from Agilent Technologies). A SphereClone ODS(2), 5μm, 250 × 4.6 mm id column was used; elution was performed by using the H_2_O (pH 3.2 by formic acid)/acetonitrile/methanol eluents and using the gradient reported in the IOC method. The injection volume was 20 μL. Identification was carried out at 280 nm. Syringic acid was used as the internal standard and the content of phenolic compounds was expressed in mg/kg of tyrosol.

The acidic hydrolysis was then applied on the hydroalcoholic extracts to evaluate the total content of free and bound tyrosol and hydroxytyrosol [23]: 300 μL of the extract was added with 300 μL of H_2_SO_4_ 1.0 M, the mixture was maintained in an oven for 2 h at 80 °C, diluted using 400 μL of water, and analyzed by HPLC–DAD. Analysis was performed using an HP1200 liquid chromatograph equipped with HP DAD (Agilent Technologies). An RP18 column, 150 × 3 mm (5 μm) Gemini (Phenomenex, Torrance, CA, USA) was used; flow rate was 0.4 mL/min. The eluents were H_2_O acidified to pH 3.2 with formic acid (A) and acetonitrile (B); the following linear solvent gradient was applied: from 95% to 70% A in 5 min, to 50% A in 5 min, to 98% B in 5 min, then a plateau of 5 min and finally to 95% in 2 min. The total time of analysis was 22 min.

### 3.6. HS–SPME–GC–MS Analysis of Volatile Organic Compounds (VOCs)

The profile of volatile compounds was analyzed by a HS–SPME–GC–MS method, recently developed and validated [24], which allowed quantification of 73 volatile organic compounds (VOCs) using 9 internal standards for area normalization. Briefly, a 4.3 g aliquot of each EVOO and 0.1 g of ISTD (internal standard) solution were weighed into 20 mL screw cap head-space vials, and immediately sealed. A tri-phase Divinylbenzene/Carboxen/Polydimethylsiloxane (DVB/CAR/PDMS) 50/30 μm SPME fiber (Agilent, Palo Alto, CA, USA) was exposed in the vial head-space under orbital shaking for 20 min, after a five-minute equilibration of sample at 45 °C. The adsorbed VOCs were desorbed in 1.7 min in the injection port (split-less mode, 260 °C) of a 6890N GC system, equipped with a MS detector (model 5975, Agilent, Palo Alto, CA, USA) and an HP–Innowax capillary column (50 m × 0.2 mm i.d., 0.4 µm film thickness) was used. The fiber was then conditioned at 260 °C for 20 min.

Oven temperature program: initial temperature 40 °C for 2 min, then 4 °C/min until 156 °C, then 10 °C/min until 260 °C. The carrier gas was helium 1.2 mL/min; temperature of ion source and transfer line was 230 °C and 250 °C, respectively. MS detector operated in scan mode in the *m*/*z* range 30–350 Th, 1500 Th/s.

Since the response of SPME fibers varies depending upon wearing and sample complexity, the addition of suitable internal standards (ISTDs) to the samples prior to analysis was necessary. An ISTD mix was selected on the basis of chemical similarity to the compounds present in the EVOOs, and contained acetic acid-d_3_, 6-chloro-2-hexanone, butanol-d_10_, 4-methyl-2-pentanol, 3-octanone, ethyl acetate-d_8_, 3,4-dimethylphenol, toluene-d_8_, and trimethylacetaldehyde in refined olive oil (IntStd). This IntStd was added to both samples and calibration standards before the analysis. Calibration lines (1st order) were built for each analyte in ranges including the concentration present in EVOO samples, using 6 diluted solutions constituted of the same amount of IntStd and different amounts of external standard. Each analyte was referred to the most suitable ISTD. The diluted standard solutions were stored in the dark at −20 °C until chromatographic analyses. Refined olive oil was used as a matrix for building the calibration lines. Mass spectra and retention times of injected authentic standards were compared with those of each peak for identification of the 73 VOCs.

### 3.7. Statistics

The analysis was carried out in triplicate and the results were expressed as mean values (mv) ± standard deviation (SD). Statistical analysis was performed using Microsoft Excel software. Significant differences between treatments were determined using one-way ANOVA followed by a *t*-test.

## 4. Conclusions

Many solutions for improving quality and oil yields have been proposed during the last decade in the olive oil sector, but only few ideas have been transferred into the market. This paper illustrates the end-user validation of the application of ultrasounds in the extra virgin olive oil extraction process. The currently used technology often considers the oil yields and the quality, associated with phenolic content and positive sensorial attributes, in antithesis. The results obtained in this research demonstrated an increase of oil yields and a reduction of the process time with a simultaneous preservation of the product quality, evaluated by validated methods. The total phenols and the total hydroxytyrosol content in EVOOs obtained from traditional milling and using the SHE system were comparable. Analogously, the sum of the molecules originating from the lipoxygenase (LOX) pathway (related to the green and fruity notes of VOOs) was not significantly affected by the application of the ultrasounds. On the contrary, the cultivar seems to affect the concentration of these molecules with highest values for Coratina cv. Anyway, no significant differences were highlighted between oils of the same cultivar obtained with or without the use of ultrasounds.

Overall, the analytical results and the collaboration with the end-users have contributed to the assessment of the machine’s usability by specific users.

The validation of the innovation and its transferability to the olive oil sector has been demonstrated by placing the machine in different geographical areas, to study its behavior towards different cultivars and different degrees of maturation. The innovative technology combines the mechanical energy of ultrasounds with the possibility of modulating the thermal exchange of olive paste. It has been demonstrated able: (i) to eliminate the malaxation step by realizing an effectively continuous process, (ii) to raise oil extraction yields recovering a part of EVOO usually lost in olive pomace, (iii) to preserve the antioxidant phenols and volatiles from the LOX pathway responsible for the fruity and green notes of virgin olive oils, (iv) to offer a sustainable plant solution able to reduce the process time and to save energy (coherently with the goals of the 2030 agenda on sustainable development), thus guaranteeing the right income to the manufacturers.

Furthermore, an added value of this work was the demonstration of the applicability of this mobile and flexible prototype machine (the SHE) in different plants produced by the world’s leading manufacturers of oil machines. The implementation of the prototype in this sector will contribute to promoting the culture of innovation as a keystone for increasing the competitiveness of the olive oil production chain by developing technological solutions such as ultrasounds to improve the production cycle and the environmental and economic sustainability of the process.

## Figures and Tables

**Table 1:** List of the analyzed extra virgin olive oils (EVOOs) produced in the different mills with (U) and without (N) the use of ultrasounds. Oils from the same mill were produced on the same days.

n°	Sample	Cultivar	Olive Mill	Crusher	MI	% of Fruit Oil Content in the Fresh Matter	% of Pomace Oil Content in the Fresh Matter
1	OM-N	Coratina	Mimì	Hammer Crusher	1	19.6 ± 0.6	7.5 ± 0.5
2	OM-U	Coratina	Mimì	Hammer Crusher	1	19.6 ± 0.6	2.0 ± 0.6
							
3	1N	Peranzana	Graco	De-stoner	3	24.8 ± 0.4	5.1 ± 0.4
4	3N	Peranzana					
5	5N	Peranzana					
6	9N	Peranzana					
7	10N	Peranzana					
8	12N	Peranzana					
9	2U	Peranzana	Graco	De-stoner	3	24.8 ± 0.4	0.7 ± 0.5
10	4U	Peranzana					
11	6U	Peranzana					
12	7U	Peranzana					
13	8U	Peranzana					
14	11U	Peranzana					
							
15	P5N	Coratina/Ogliarola	Frantolio	Hammer Crusher	4	21.0 ± 0.4	1.8 ± 0.4
16	P6N	Coratina/Ogliarola					
17	P3U	Coratina/Ogliarola	Frantolio	Hammer Crusher		21.0 ± 0.4	
18	P4U	Coratina/Ogliarola			4		0.7 ± 0.2
19	P7U	Coratina/Ogliarola					
							
20	C4N	Cellina di Nardò	Frantolio	Hammer Crusher	6	14.8 ± 0.3	1.6 ± 0.4
21	C3U	Cellina di Nardò	Frantolio	Hammer Crusher	6		
22	C5U	Cellina di Nardò				14.8 ± 0.3	0.8 ± 0.3
23	C6U	Cellina di Nardò					

**Table 2:** Maturity index (MI) classification groups of olives based on skin and flesh color.

Maturity Index Group	0	1	2	3	4	5	6	7
	Skin color deep green	Skin color yellow-green	Skin color with <half the fruit surface turning red, purple, or black	Skin color with >half the fruit surface turning red, purple, or black	Skin color all purple or black with all white or green flesh	Skin color all purple or black with <half the flesh turning purple	Color all purple or black with >half the flesh turning purple	Skin color all purple or black with all the flesh purple to the pit

**Table 3:** Distribution of the different groups of volatiles produced during the lipoxygenase cascade (first 5 rows) and linked to oxidative defects (last 3 rows) in oils obtained with (U) and without (N) the use of SHE. For each couple of oils, no differences were highlighted as indicated by the same letter (a).

VOC	1N–12N		P3U–P7U		C3U–C6U		OMN–OMU	
	U	N	U	N	U	N	U	N
LOX C5	0.25 a	0.25 a	0.47 a	0.53 a	0.15 a	0.14 a	1.61 a	1.46 a
LOX C6 aldehydes	7.98 a	8.24 a	11.98 a	12.06 a	5.22 a	5.16 a	34.24 a	34.78 a
LOX C6 esters	2.91 a	2.89 a	0.06 a	0.05 a	0.32 a	0.29 a	0.07 a	0.07 a
LOX C6 alcohols	0.77 a	0.76 a	1.36 a	1.77 a	0.70 a	0.55 a	1.63 a	1.77 a
Total LOX	11.92 a	12.14 a	13.87 a	14.41 a	6.40 a	6.14 a	37.55 a	38.07 a
saturated C7–C10 aldehydes	<0.01 a	<0.01 a	0.29 a	0.30 a	0.01 a	0.01 a	0.15 a	0.18 a
mono-unsaturated C7–C10 aldehydes	0.02 a	0.02 a	<0.01 a	<0.01 a	0.05 a	0.04 a	<0.01 a	<0.01 a
di-unsaturated C7–C10 aldehydes	<0.01 a	<0.01 a	0.24 a	0.26 a	0.03 a	0.02 a	0.12 a	0.13 a

## Supplementary Materials

The following are available online, Figure S1: Spider web plot obtained by sensorial analysis of two samples, obtained by the same batch of olives processed with (green in the plot) or without (yellow) the use of ultrasounds.
