# Supplementary material for: Implementation of the Sono-Heat-Exchanger in the Extra Virgin Olive Oil Extraction Process: End-User Validation and Analytical Evaluation
Source: Molecules. 2019 Jun 27;24(13):2379. doi: 10.3390/molecules24132379 (PMC6651205; doi:10.3390/molecules24132379)

**Supplementary material**

**Figure S1**. Spider web plot obtained by sensorial analysis of two samples, obtained by the same batch of olives processed with (green in the plot) or without (yellow) the use of ultrasounds

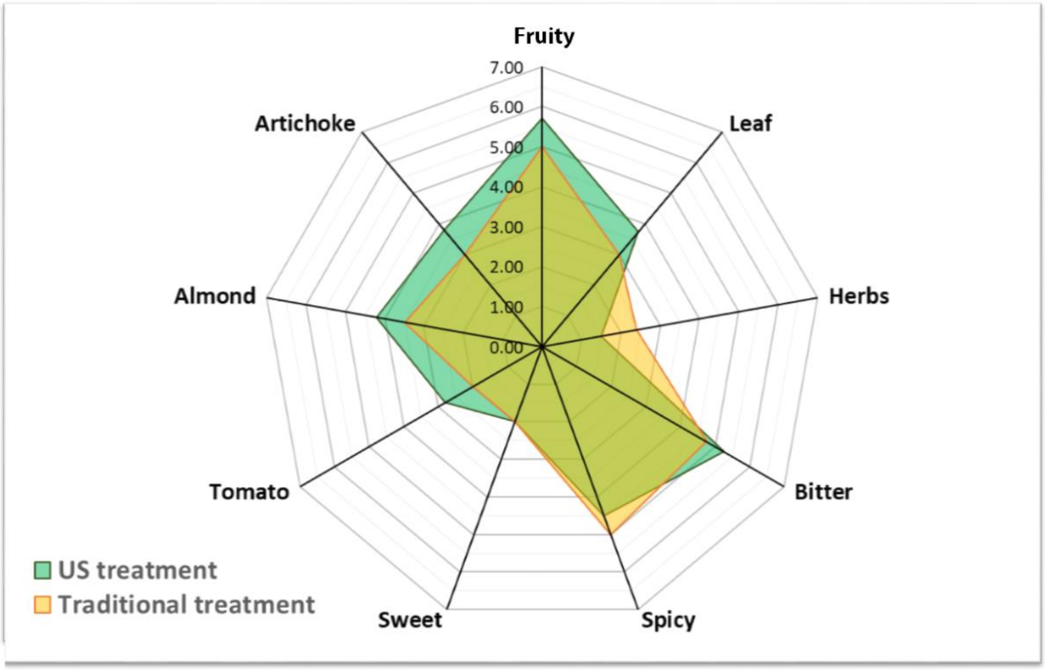

Supplement: Supplementary file 1 [file molecules-24-02379-s001.pdf]
